# Using stable isotopes analysis to understand ontogenetic trophic variations of the scalloped hammerhead shark at the Galapagos Marine Reserve

**DOI:** 10.1371/journal.pone.0268736

**Published:** 2022-06-10

**Authors:** Florencia Cerutti-Pereyra, Pelayo Salinas-De-León, Camila Arnés-Urgellés, Jennifer Suarez-Moncada, Eduardo Espinoza, Leandro Vaca, Diego Páez-Rosas

**Affiliations:** 1 Charles Darwin Research Station, Charles Darwin Foundation, Puerto Ayora, Galapagos Islands, Ecuador; 2 Save Our Seas Foundation Shark Research Center and Guy Harvey Research Institute, Nova Southeastern University, Dania Beach, Florida, United States of America; 3 Galápagos National Park Directorate, Puerto Ayora, Galápagos, Ecuador; 4 Migramar Marine Research and Conservation Network, Olema, California, United States of America; 5 Galapagos Science Center, Universidad San Francisco de Quito, Puerto Baquerizo Moreno, Galápagos, Ecuador; University of California Davis, UNITED STATES

## Abstract

Changes in life-history requirements drive trophic variations, particularly in large marine predators. The life history of many shark species is still poorly known and understanding their dietary ontogeny is a challenging task, especially for highly migratory species. Stable isotope analysis has proven as a useful method for examining the foraging strategies of sharks and other marine predators. We assessed the foraging strategies and ontogenetic changes of scalloped hammerhead sharks, *Sphyrna lewini*, at Galapagos Marine Reserve (GMR), by analysing δ^13^C and δ^15^N signatures in different maturity stages. Our isotopic results suggest ontogenetic shifts in resource use between sub-adult and adult stages, but not between adult and juvenile stages. Carbon isotopic signatures found in the juvenile stage were enriched in contrast to sub-adults (~0.73‰) suggesting a combination of the maternal input and the use of coastal resources around the Galapagos Islands. Adult female sharks also showed enrichment in δ^13^C (~0.53‰) in comparison to sub-adult stages that suggest feeding in high primary productivity areas, such as the GMR. This study improves the understanding of the trophic ecology and ontogenetic changes of a highly migratory shark that moves across the protected and unprotected waters of the Eastern Tropical Pacific.

## Introduction

The global decline of large predators, such as sharks, has had major impacts on many marine food webs [1, 2], since most shark species are primary or secondary predators controlling trophic relationships and energy flow in the ecosystems they occupy [3]. Understanding foraging strategies of such predators that are highly mobile, provides key information about their basic ecology and migratory patterns [4, 5]. These life history aspects are very valuable when implementing management strategies aimed at reducing ongoing global shark population declines [6, 7].

The trophic ecology of sharks has been traditionally studied using gut content analysis from fisheries landings [8, 9]. However, this analysis can be challenging when studying threatened large migratory species, particularly in areas where their capture is not permitted, such as fully-protected marine reserves [10]. Stable isotopes of carbon (δ^13^C) and nitrogen (δ^15^N) in animal tissues are a non-lethal alternative that can provide retrospective information on foraging ecology, since the isotopic composition of the predator reflects the assimilated prey information over time [4, 11]. The isotopic signatures of marine primary producers vary depending on biogeochemical and oceanographic processes. Such differences get spread through local food webs resulting in consumers with isotopic signatures resembling the food webs they feed on [12, 13].

The scalloped hammerhead shark (*Sphyrna lewini*) is distributed globally in tropical and semitropical waters [14]. The oceanic islands of the Eastern Tropical Pacific (ETP) harbour some of the largest aggregations of this species [15–17] that have become a significant tourist attraction and support lucrative SCUBA diving industries [18, 19]. *Sphyrna lewini* show seasonal aggregations and migratory patterns within the ETP, covering thousands of miles in their migrations. These regional movements include unprotected waters between the oceanic Marine Protected Areas (MPA) of the Galapagos (Ecuador), Malpelo (Colombia) and Isla del Coco (Costa Rica) where domestic and international fishing fleets operate [20–22]. Despite its ecological and economic importance, this species is heavily fished and caught as bycatch [23, 24], which has greatly decreased their populations [25–29]. Such alarming decrease has led to its listing as ‘Critically Endangered’ (CR) by the IUCN Red List in 2019 and its inclusion in the Annex II of the Convention on International Trade in Endangered Species (CITES) [24, 30].

*Sphyrna lewini* shows sexual segregation in the ETP, with aggregations found in waters around oceanic island skewed towards large females which likely migrate to coastal waters to give birth [20, 21, 31, 32] after mating in open waters [33]. In contrast, male sharks are thought to migrate more widely in open waters [34, 35]. Juvenile *S*. *lewini* sharks are commonly found in shallow waters, which function as nursery grounds along the ETP, and are often caught in coastal artisanal fisheries [36–40]. Although the life history of *S*. *lewini* sub-adult stage is noticeably less understood than adult and juvenile stages, it is known that sub-adult female sharks migrate from nursery areas sooner than sub-adult male sharks [31, 41]. However, sub-adult sharks are less commonly found in aggregations where large female sharks are dominant [17, 42] and this apparent disappearance of the transitory sub-adult stage hinders the understanding of this highly migratory species’ ecology.

The Galapagos Marine Reserve (GMR) has afforded over 20 years of protection from industrial fishing for many species of sharks, including *S*. *lewini* [15, 17, 43], though many endangered species are often caught as bycatch in artisanal longline fisheries [44]. Studies on residency and migratory patterns using telemetry show that *S*. *lewini* is semi-resident in oceanic islands of the ETP and has complex movement patterns around them. During daytime, *S*. *lewini* stays close to the islands and disperses into deep and open water during night-time, likely to forage on deep-water prey [17, 22, 45, 46]. Stomach content analyses from several coastal locations across the ETP suggest this species forages on a variety of prey. Generally, cephalopods are the main prey for adult sharks, while crustaceans and bony fish are the primary prey of juvenile sharks [40, 47, 48]. Particularly, adult female hammerhead sharks seem to prefer *Octopus* spp. and *Histioteuthis* spp., while the squids *Dosidicus gigas* and *Mastigoteuthis* spp. are the most common prey in juvenile individuals [40, 49]. Despite this knowledge of *S*. *lewini* in Ecuadorian waters, nothing is known about their trophic ecology at the GMR which is a concern given ongoing population declines in the region [25, 26].

Here, we present the first study of foraging strategies of *S*. *lewini* at the GMR, establishing a baseline of the trophic ecology of an endangered species inhabiting an almost-pristine biodiversity hotspot. We hypothesized there would be differences in the isotopic niches of *S*. *lewini* throughout its ontogeny as a result of variance in foraging strategies and environmental variability in the region.

## Materials and methods

### Ethics statement

This research was approved by the Galapagos National Park Directorate (GNPD) on the research permits granted to Pelayo Salinas-de-León from the Charles Darwin Foundation (CDF) (Research Permits: PC-27-17, PC-46-18 and PC-53-19), and Diego Páez-Rosas from the University San Francisco de Quito (USFQ) (PC-24-17, PC-69-18 and PC-86-19). The methods described here were reviewed and approved by the GNPD, CDF and USFQ committees responsible for assessing animal welfare in research activities.

### Study site

**Fig 1:**
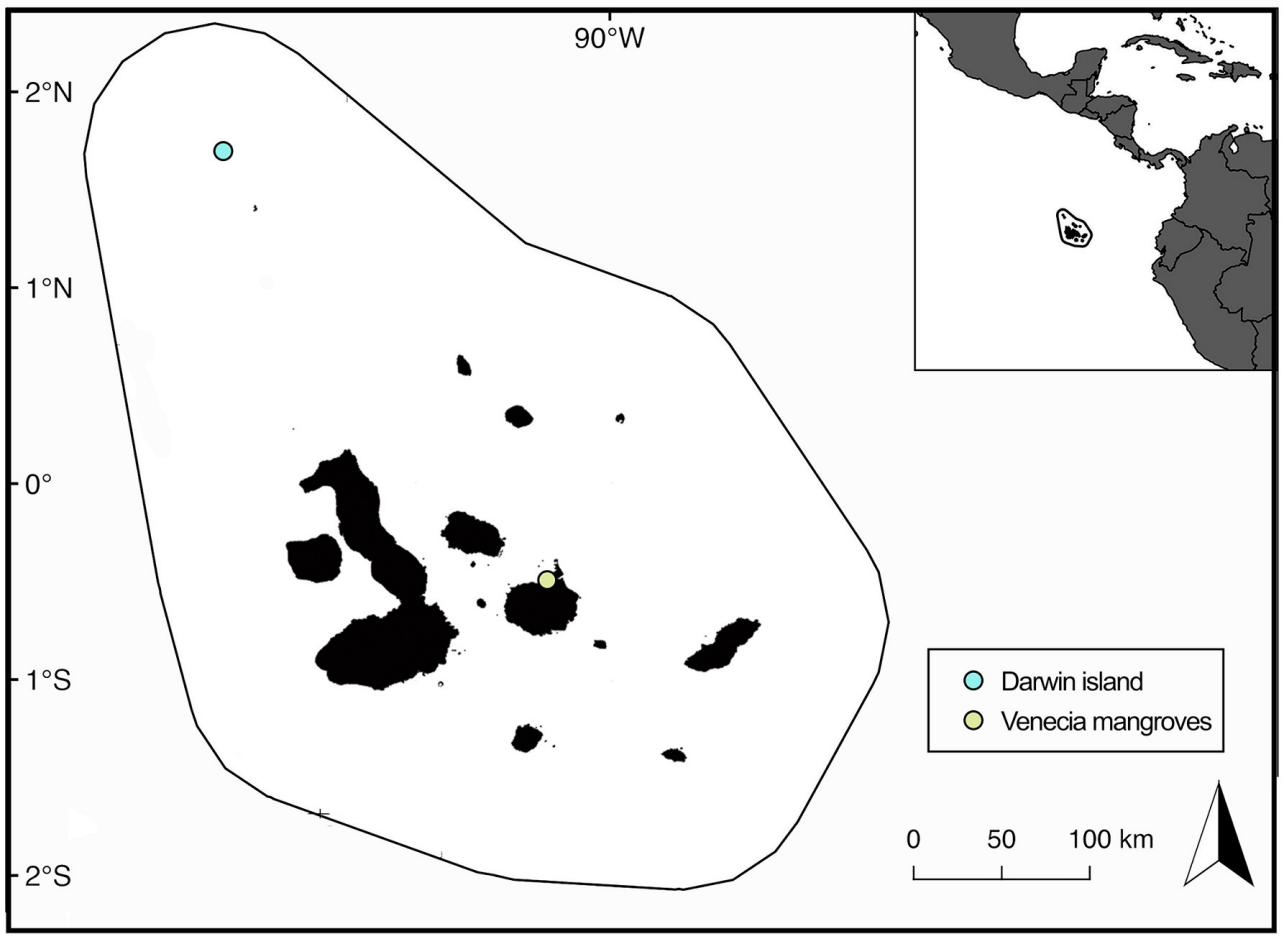
Sampling sites for *Sphyrna lewini* stable isotope analysis at the Galapagos Marine Reserve. Samples of adults and sub-adult sharks were collected around Darwin Island. Samples of juvenile sharks were collected at Venecia mangroves, north of Santa Cruz Island Map was obtained from NOAA coastal extractor: https://www.ngdc.noaa.gov/mgg/shorelines/ [55].

The Galapagos archipelago (Fig 1) is located approximately 1,000 km west from mainland Ecuador and comprises 15 major islands [50]. The archipelago lays within an upwelling system due to the convergence of major ocean currents that characterize the ETP [51–53]. Variations in the strength of these currents throughout the year produce two distinct seasons: the warm season from January to May and the cool season from July to November, while June and December are considered transitional months [51, 54].

### Sample collection

Skin samples from large pregnant female sharks (>230 cm TL, n = 64) and the smallest individuals (<150, n = 29, referred to as subadults hereafter) that could be located among the large groups aggregating at Darwin Island [15] were collected during August 2017 and October 2019. These samples were taken using sterile 6-mm biopsy punches to remove ~10 mg of skin tissue close to the first dorsal fin, using a biopsy probe (Pneudart Inc., USA) attached to a Hawaiian sling pole while freediving at Darwin Island (Fig 1). During May 20178 and August 2019, skin samples from the first dorsal fin (fin clips) from juvenile sharks (44 to 89 cm TL, n = 33) were collected in shallow mangrove bays of Santa Cruz Island. For such sampling, juvenile sharks were considered when the size is ≤100 cm TL and had a visible and closed umbilical cord wound [56, defined as USS level 4 by 57]. Juvenile shark catch-and-release procedures were based on a standard protocol for monitoring live sharks in shallow bays developed by the GNPD [58]. All collected samples were stored at -20°C.

### Sample processing

In the laboratory, shark skin samples were rinsed with deionized water to eliminate residues that could alter their isotopic signatures. Samples were placed in glass vials, which were previously treated for 24 h with a chromic acid mixture prepared from sulfuric acid and potassium dichromate [59]. Samples were then dried in a desiccator at 80°C for 12 h to remove all moisture and a lipid-extracted protocol was applied via three sequential 24 h soaks in a 2:1 chloroform:methanol solution, then rinsed with deionized water and dried again [5]. This process was applied because lipids incorporate less δ^13^C (i.e. more negative δ^13^C) which, in sufficiently large quantities, could negatively skew the results of δ^13^C [60]. Samples were homogenized with an agate mortar to obtain a very fine powder, of which ∼1 mg was weighed by means of an analytical microbalance with a precision of 0.001 mg and transferred into a tin capsule for isotopic analysis.

Stable isotope ratios of δ^13^C and δ^15^N were determined by a PDZ Europa 20–20 continuous flow isotope-ratio mass spectrometer (Sercon Ltd., Cheshire, UK) at the Stable Isotope Facility of the University of California, Davis (CA, USA). The precision of the isotopic analyses was 0.15, based on internal reference samples. Ratios of heavy to light isotopes were expressed in δ notation using the equation: δX = [(R_sample_/R_standard_)-1]*1000, where X is the heavy isotope, R_sample_ is the ratio of heavy to light isotope in the sample, and R_standard_ is the ratio of heavy to light isotope in the reference standard. The standard reference material for carbon was Pee Dee Belemnite (PDB) and for nitrogen was atmospheric N_2_. Units are part per thousand (per mil, ‰). Within-run analytical precision was estimated via analysis of two proteinaceous internal reference materials, which was ±0.2‰ for both δ^13^C and δ^15^N values. We also measured the weight percentage of carbon and nitrogen concentration of each sample and used the C/N ratio as a proxy of evaluating the extent of lipid content that is likely to affect the results of δ^13^C values [61].

#### Data analysis

Data were tested for normality and homoscedasticity using the Shapiro-Wilk and Levene test, respectively. The statistical significance in δ^13^C and δ^15^N values by life stage and by years was determined using parametric (t-test and ANOVA tests) or non-parametric (Mann-Whitney and Kruskal-Wallis test) analysis and reported at a significance level of p < 0.05. All statistical analyses were performed using R software. Bayesian standard ellipse areas (SEAb) were used to estimate isotopic niche width and overlap among samples collected using the package SIBER (Stable Isotope Bayesian Ellipses in R) [62]. This Bayesian method provides a measure of the isotopic niche area at the population level, expressed as the SEAb in units of area (‰^2^), the niche area containing 95% of the data for each group was chosen. We used Monte Carlo simulations to correct the bivariate ellipses (δ^13^C and δ^15^N) surrounding the data points in the 95% confidence interval for the distributions of both stable isotopes [62]. These Bayesian ellipses are corrected using posteriori randomly replicated sequences (SEAc = standard ellipse area correction) [62]. The magnitude of the isotopic overlap (‰^2^) were estimated using the estimations of the ellipses via maximum likelihood methods [62].

## Results

### Isotopic comparison between maturity stages

**Fig 2:**
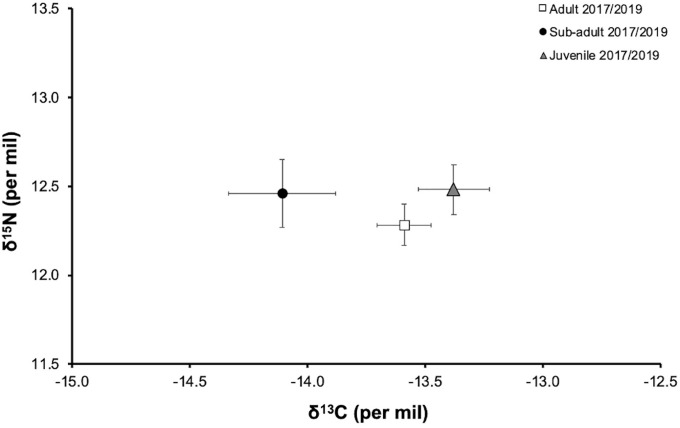
Isotopic values of δ^13^C and δ^15^N (mean +SE in ‰) of adult, sub-adult and juvenile *Sphyrna lewini* at the Galapagos Marine Reserve.

The δ^13^C values of *S*. *lewini* varied between -16.58‰ and -11.79‰, with an average of -13.38 (-14.96 to -11.79‰) for juvenile, -14.11 (-16.58 to -13.19‰) for sub-adult, and -13.59‰ (-14.93 to -12.54 ‰) for adult sharks. While the range for δ^15^N values varied between 11.03‰ and 13.91‰, with an average of 12.48‰ (10.77 to 13.91‰) for juvenile, 12.46‰ (10.77 to 13.69‰) for sub-adult, and 12.28‰ (10.77 to 15.05‰) for adult sharks (Table 1, S1 Appendix). The C:N values ranged from 2.65 to 3.44 with an average of 2.9 + 0.17 (Table 1), falling within the theoretical range established for the assimilation of protein from a predator’s diet [62, 63]. The comparison showed that juvenile sharks have slightly enriched ^13^C values compared to adult and sub-adult stages. All three maturity stages had a similar trophic level, although adults had slightly depleted δ^15^N. The comparison also showed that sub-adult sharks had wider values for both δ^13^C and δ^15^N (Table 1 and Fig 2).

**Table 1:** Summary of isotopic values of δ^13^C and δ^15^N (mean +SD ‰) and C:N ratio (for *Sphyrna lewini* at the Galapagos Marine Reserve (TL = total length in cm).

Year	Maturity stage	Site	n	Size	δ13C (‰)	δ15N (‰	Ratio C:N (Mean ± SD)
				Range (TL, cm)	Mean (+ SD)	Mean (+ SD)	
2017	Sub-adults	Darwin Island	13	<150	-13.86 (0.66)	12.43 (0.61)	2.85 (0.14)
2017	Adults	Darwin Island	30	>230	-13.49 (0.48)	12.32 (0.60)	2.78 (0.13)
2017	Juveniles	Santa Cruz	14	58–82 (x = 68)	-13.09 (0.79)	12.54 (0.49)	3.01 (0.06)
2019	Juveniles	Santa Cruz	19	44–89 cm (x = 66)	-13.67 (0.39)	12.42 (0.66)	2.94 (0.10)
2019	Sub-adults	Darwin Island	16	<150	-14.36 (1.09)	12.50 (0.86)	2.96 (0.18)
2019	Adults	Darwin Island	34	>230	-13.69 (0.19)	12.25 (0.71)	3.04 (0.19)

The δ^15^N values did not vary when comparing years in all maturity stages (t-test adults p = 0.68, sub-adults p = 0.80, juveniles p = 0.56) but there was a significant difference in δ^13^C values between years for juvenile sharks (Mann-Whitney test adults p = 0.122, sub-adults p = 0.30, juveniles p = 0.001). No significant difference was found in δ^15^N values among maturity stages (ANOVA test p = 0.28), although juveniles showed the highest (enriched) values and adults showed the lowest (depleted). For δ^13^C values, sub-adult sharks had the lowest (depleted) values and showed a significant difference (Kruskal-Wallis test p = 0.01) when compared to adult and juvenile sharks (Table 2).

**Table 2:** Isotopic values of δ^13^C of adult, sub-adult, and juvenile *S*. *lewini* in the Galapagos Marine Reserve.

Maturity stage	Adult	Sub-adult	Juvenile
Adult	X		
Sub-adult	**0.024**	X	
Juvenile	1.000	**0.006**	X

Significant differences (Kruskal-Wallis test, p < 0.05) are shown in bold.

### Niche breadth and isotopic overlap

**Fig 3:**
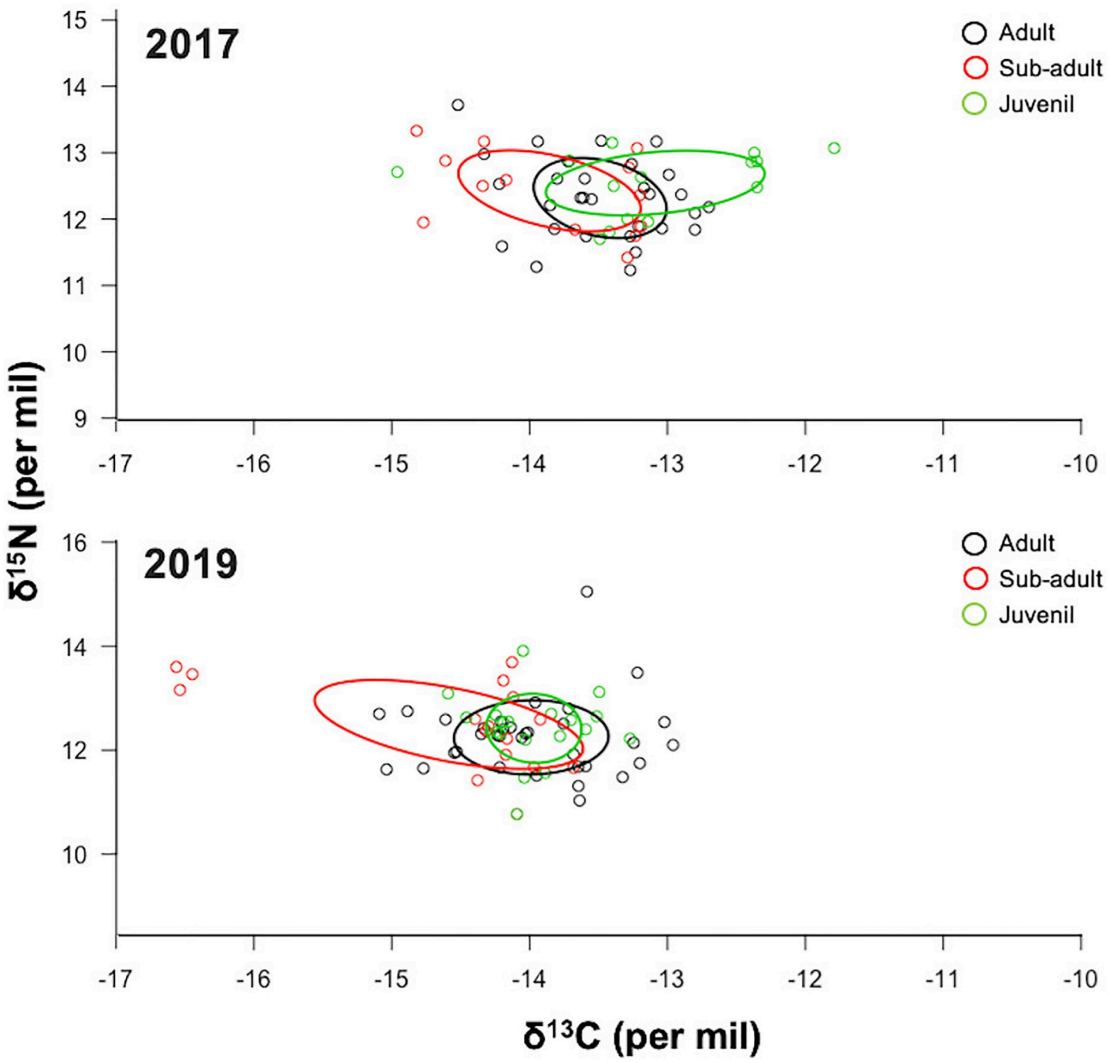
Isotopic niche area (δ^13^C and δ^15^N values) of adult, sub-adult, and juvenile *Sphyrna lewini* in the Galapagos Marine Reserve during 2017–2019. The ellipse areas show the degree of trophic niche overlap among maturity stages.

The corrected standard ellipse area (SEAc) showed a similar isotopic niche in adult and juvenile stages, while sub-adult sharks had a larger isotopic niche in both years (Fig 3 and Table 3). The ellipses of sub-adult (SEAc = 1.24‰, credibility interval of 0.75–1.73‰) and juvenile sharks (SEAc = 1.26‰, credibility interval of 0.88–1.64‰) had a low overlap in 2017–2018. While the overlap area of adult and sub-adult sharks in 2017–2018 (0.57) was significant representing 80.1% of the ellipse surface of adult sharks and 52.5% of sub-adult sharks. The juvenile sharks’ ellipse in 2017 was larger and show a low isotopic overlap relative to adult and sub-adult sharks (0.44 and 0.38 respectively, Fig 3). In 2019, the ellipse of sub-adult (SEAc = 3.02‰, credibility interval of 2.17–3.87‰) and adult sharks (SEAc = 1.64‰, credibility interval of 0.96–2.32‰) showed a low overlap that represented 74.1% of the ellipse surface of sub-adult and the 37.5% of the ellipse surface of adult sharks. A significant overlap was observed between adult and juvenile sharks in 2019 (0.50, Fig 3).

**Table 3:** Degree of isotopic niche overlap between maturity stages of *Sphyrna lewini* in the Galapagos Marine Reserve.

Maturity stage	Adult	Sub-adult	Juvenile
**2017**			
Adult	X		
Sub-adult	**0.578**	X	
Juvenile	0.440	0.384	X
**2019**			
Adult	X		
Sub-adult	0.361	X	
Juvenile	**0.504**	0.244	X

The degree of trophic niche overlap where a value close to 1 (shown in bold) indicates a large overlap between trophic niches.

## Discussion

This is the first study addressing the trophic ecology of *Sphyrna lewini* at the Galapagos Marine Reserve. Our results reveal ontogenetic changes from sub-adult to adult stages as observed in other areas across the ETP [31, 32, 41] and suggest a wide range of resource use by this migratory shark through its lifetime [47].

Sub-adult *S*. *lewini* at the GMR show a wider pattern of resource use than adult and juvenile sharks and had a niche overlap with both adult and juvenile stages. This mixture of isotopic signatures is likely the result from an ontogenetic change in movements of sub-adults, accompanied by use of a wider variety of food sources during this maturity stage. Coastal ecosystems have enriched δ^13^C signatures compared to pelagic environments due to the high primary production that subsidize carbon derived from benthic macrophytes in coastal zones [64]. Additionally, the majority of deep ocean areas are homogenous in terms of nitrogen sources (nitrates), so the δ^15^N values are relatively higher in deeper waters than shallower areas where the primary source is the atmospheric nitrogen [65, 66]. The depleted δ^13^C and enriched δ^15^N signatures found in sub-adult sharks suggest they are feeding in oceanic and deeper waters. Particularly, the higher values of δ^15^N in sub-adult *S*. *lewini* suggest that sharks are moving from coastal shallow waters to oceanic and deeper waters through this life stage as observed also in the Mexican Pacific populations [31, 32, 41]. These ontogenetic shifts in resource use where adult and juvenile sharks show coastal strategies, and sub-adult sharks a more pelagic behaviour have also been observed in other species of sharks in Ecuadorian waters, such as smooth hammerhead sharks (*S*. *zygaena*) [67] and blue sharks (*Prionace glauca*) [68].

Juvenile sharks showed generally enriched values of δ^13^C compared to sub-adult and adult sharks, suggesting the use of coastal environments as nursery areas as reported in other locations of the Pacific Ocean [57, 69]. Surprisingly, juvenile sharks sampled in May 2018 were significantly enriched in δ^13^C signatures compared to August 2019. The integration of isotopic composition over several months due to the isotopic skin-diet fractionation [3–6 months in elasmobranchs 70] show variations associated with the seasonal changes in marine productivity commonly found within the GMR. The juvenile sharks sampled in May 2018, are likely reflecting the diet consumed during the cold season (October-November) characterized by more productive waters [71], whereas juvenile sharks sampled in August 2019 would reflect foraging during the hot season (February-March) which is characterized by less nutrient-rich waters [51, 54].

All juvenile sharks sampled had a clearly visible and closed umbilical wound indicating they were younger than a year old [57]. The slight enrichment of δ^15^N signatures in juvenile sharks could be explained by a reminiscence from the mother’s isotopic values assimilated through maternal transfer processes [48, 72, 73]. As pseudo-placental sharks, female *S*. *lewini* transfer nutrients directly to their offspring through a yolk-sac [74], thus the isotopic signatures of juvenile sharks are likely a mixture of inherited isotopic signatures resulting in a similar isotopic signature to adult females. Importantly, while juvenile blacktip sharks (*Carcharinus limbatus*) have been commonly recorded in mangrove bays within the GMR by the GNPD shark monitoring program [58, 75, 76], juvenile *S*. *lewini* sharks have been less frequently documented in the central islands of the GMR before this sampling event [77].

The enriched signatures of δ^13^C and the overlap of isotopic niche between adult female and juvenile sharks suggest that both maturity stages are exploiting similar environments. All adult sharks sampled in this study were pregnant females that occur in large aggregations at Darwin Island and show seasonal residency between June to March [15, 17, 22]. There is an increase in blooms from the equator to the Galapagos Islands caused by the divergence in equatorial blooms associated to the wind and equatorial sub-stream intensity [78]. These increase of primary productivity levels generates an increase in phytoplankton biomass and, therefore, its respiration capacity, resulting in high CO_2_ absorption from the aqueous environment [79, 80]. Phytoplankton blooms tend to enrich the ^13^C signal, even though the high levels of photosynthesis cause a rapid use and decrease of CO_2_ (enriched in ^12^C). In this process, there is discrimination towards ^13^C resulting in the enrichment of δ^13^C values in primary producers and higher trophic levels [79, 80]. The enriched signatures of δ^13^C in both juveniles and adult female sharks suggest a pattern of residence for adult female *S*. *lewini* at the GMR where there are high levels of productivity that facilitate their permanence [51, 54].

Telemetry studies of *S*. *lewini* at the GMR have shown that females at Wolf and Darwin Islands move within the GMR and migrate between oceanic islands in the ETP [17, 22], while genetic studies show that *S*. *lewini* found in Malpelo Island are related to neonates found in coastal nurseries in mainland Colombia [34]. The presence of various species of sharks in juvenile stages including the *S*. *lewini* highlights the importance of Galapagos mangrove areas for recruitment of key marine fauna in the GMR [58, 76, 81] and highlights the importance of better understanding coastal-oceanic linkages across the ETP maintained by this highly migratory species [39, 77, 82].

## Conclusions

The isotopic signatures of *S*. *lewini* at the GMR indicate an ontogenetic shift of resource use in sub-adult and adult sharks as observed in other areas within the ETP. The δ^13^C and δ^15^N signatures range in adult and sub-adult sharks suggest foraging on a variety of prey and ecosystems: coastal and pelagic, shallow and deep waters. These changes coincide with the potential residency of adult female sharks and the long-distance migrations between islands and mainland by sub-adult sharks found by previous studies in the ETP using gut content, microchemistry, and telemetry.

Understanding the foraging strategies of a migratory shark that uses a variety of sources throughout its lifetime and in an area with particular oceanographic conditions such as the GMR can be quite challenging. Identifying prey items within the reserve will help better understanding the trophic ecology of this species and characterize the isoscape over which these sharks move. Combining stable isotope analysis with telemetry data would also be useful to monitor long-term *S*. *lewini* feeding patterns in relation to variations in prey availability and oceanographic conditions. Understanding how each life stage uses resources and how coastal and oceanic aggregations are interconnected will contribute to guide national management plans, regional conservation initiatives, and international treaties to better protect a critically endangered, highly migratory predator whose ontogenetic migratory patterns occur within the jurisdictional waters of several countries.

## Supporting information

S1 Appendix Values of *S*. *lewini* N and C stable isotopes at the Galapagos Islands. (XLSX)

## References

[1] Baum JK, Worm B. Cascading top-down effects of changing oceanic predator abundances. J Anim Ecol. 2009;78: 699–714. doi: 10.1111/j.1365-2656.2009.01531.x 19298616

[2] Heithaus MR, Frid A, Wirsing AJ, Worm B. Predicting ecological consequences of marine top predator declines. Trends Ecol Evol. 2008;23: 202–210. doi: 10.1016/j.tree.2008.01.003 18308421

[3] Myers RA, Baum JK, Shepherd TD, Powers SP, Peterson CH. Cascading effects of the loss of apex predatory sharks from a coastal ocean. Science. 2007;315: 1846. doi: 10.1126/science.1138657 17395829

[4] Graham BS, Koch PL, Newsome SD, McMahon KW, Aurioles D. Using Isoscapes to Trace the Movements and Foraging Behavior of Top Predators in Oceanic Ecosystems. In: West JB, Bowen GJ, Dawson TE, Tu KP, editors. Isoscapes: Understanding movement, pattern, and process on Earth through isotope mapping. Dordrecht: Springer Netherlands; 2010. pp. 299–318. doi: 10.1007/978-90-481-3354-3_14

[5] Páez-Rosas D, Galván-Magaña F, Baque-Menoscal J, Tripp-Valdez A, Fischer C, Hearn A. Trophic preferences of three pelagic fish inhabiting the Galapagos Marine Reserve: ecological inferences using multiple analyses. Environ Biol Fishes. 2020.

[6] Hussey NE, MacNeil MA, Olin JA, McMeans BC, Kinney MJ, Chapman DD, et al. Stable isotopes and elasmobranchs: tissue types, methods, applications and assumptions. J Fish Biol. 2012;80: 1449–1484. doi: 10.1111/j.1095-8649.2012.03251.x 22497393

[7] Roff G, Brown CJ, Priest MA, Mumby PJ. Decline of coastal apex shark populations over the past half century. Commun Biol. 2018;1: 1–11. doi: 10.1038/s42003-018-0233-1 30564744 PMC6292889

[8] Torres-Rojas YE, Hernández-Herrera A, Galván-Magaña F, Alatorre-Ramírez VG. Stomach content analysis of juvenile, scalloped hammerhead shark *Sphyrna lewini* captured off the coast of Mazatlán, Mexico. Aquat Ecol. 2010;44: 301–308. doi: 10.1007/s10452-009-9245-8

[9] Polo-Silva C, Newsome SD, Galván-Magaña F, Grijalba-Bendeck M, Sanjuan-Muñoz A. Trophic shift in the diet of the pelagic thresher shark based on stomach contents and stable isotope analyses. Mar Biol Res. 2013;9: 958–971. doi: 10.1080/17451000.2013.793802

[10] Salinas-De-León P, Fierro-Arcos D, Suarez-Moncada J, Proaño A, Guachisaca-Salinas J, Páez-Rosas D. A matter of taste: Spatial and ontogenetic variations on the trophic ecology of the tiger shark at the Galapagos Marine Reserve. PLoS One. 2019;14: 0–20. doi: 10.1371/journal.pone.0222754 31539419 PMC6754146

[11] Trueman CN, St John Glew K. Isotopic Tracking of Marine Animal Movement. In: Hobson K, Wassenaar LI, editors. Tracking animal migration with stable isotopes. Academic Press; 2019. p. 269.

[12] Fry B, Sherr EB. δ13C measurements as indicators of carbon flow in marine and freshwater ecosystems. Cotributions to Mar Sci. 1984;27: 13–47.

[13] Hobson K, Ambrose WG, Reanud P. Sources of primary production, benthic-pelagic coupling, and trophic relationships within the Northeast Water Polynia: insights form 13C and 15N analysis. Mar Ecol Prog Ser. 1995;128: 1–10.

[14] Compagno LJ V. FAO species identification guide for fishery purposes. The living marine resources of the Western Central Pacific. FAO. Rome, Italy; 1999.

[15] Salinas de León P, Acuña-Marrero D, Rastoin E, Friedlander AM, Donovan MK, Sala E. Largest global shark biomass found in the northern Galapagos Islands of Darwin and Wolf. PeerJ. 2016;4: e1911. doi: 10.7717/peerj.1911 27190701 PMC4867720

[16] Friedlander AM, Zgliczynski BJ, Ballesteros E, Aburto-Oropeza O, Bolaños A, Sala E. The shallow-water fish assemblage of Isla del Coco National Park, Costa Rica: Structure and patterns in an isolated, predator-dominated ecosystem. Rev Biol Trop. 2012;60: 321–338.

[17] Hearn A, Ketchum J, Klimley AP, Espinoza E, Peñaherrera C. Hotspots within hotspots? Hammerhead shark movements around Wolf Island, Galapagos Marine Reserve. Mar Biol. 2010;157: 1899–1915. doi: 10.1007/s00227-010-1460-2 24391250 PMC3873083

[18] Lynham J, Costello C, Gaines S, Sala E. Economic valuation of marine- and shark-based tourism in the Galapagos Islands: Report to the Galapagos National Park. 2015.

[19] Sibaja-Cordero JA. Tendencias espacio-temporales de los avistamientos e fauna marina en los buceos turisticos (Isla del Coco, Costa Rica). Rev Biol Trop. 2008;56: 113–132.18624230

[20] Nalesso E, Hearn A, Sosa-Nishizaki O, Steiner T, Antoniou A, Reid A, et al. Movements of scalloped hammerhead sharks (*Sphyrna lewini*) at Cocos Island, Costa Rica and between oceanic islands in the Eastern Tropical Pacific. PLoS One. 2019;14. doi: 10.1371/journal.pone.0213741 30861051 PMC6413943

[21] Bessudo S, Soler GA, Klimley AP, Ketchum JT, Hearn A, Arauz R. Residency of the scalloped hammerhead shark (*Sphyrna lewini*) at Malpelo Island and evidence of migration to other islands in the Eastern Tropical Pacific. Environ Biol Fishes. 2011;91: 165–176. doi: 10.1007/s10641-011-9769-3

[22] Ketchum JT, Hearn A, Klimley AP, Peñaherrera C, Espinoza E, Bessudo S, et al. Inter-island movements of scalloped hammerhead sharks (*Sphyrna lewini*) and seasonal connectivity in a marine protected area of the eastern tropical Pacific. Mar Biol. 2014;161: 939–951. doi: 10.1007/s00227-014-2393-y

[23] Martínez-Ortiz J, Aires-Da-silva AM, Lennert-Cody CE, Maunderxs MN. The ecuadorian artisanal fishery for large pelagics: Species composition and spatio-temporal dynamics. PLoS One. 2015;10: 1–29. doi: 10.1371/journal.pone.0135136 26317751 PMC4552643

[24] Rigby CL, Dulvy NK, Barreto R, Carlson J, Fernando D, Fordham S, et al. *Sphyrna lewini*. The IUCN Red List of Threatened Species 2019: e.T39385A2918526. Downloaded on 20 April 2020. 2019.

[25] Peñaherrera-Palma C, van Putten I, Karpievitch Y V., Frusher S, Llerena-Martillo Y, Hearn AR, et al. Evaluating abundance trends of iconic species using local ecological knowledge. Biol Conserv. 2018;225: 197–207. doi: 10.1016/j.biocon.2018.07.004

[26] White ER, Myers MC, Flemming JM, Baum JK. Shifting elasmobranch community assemblage at Cocos Island-an isolated marine protected area. Conserv Biol. 2015;29: 1186–1197. doi: 10.1111/cobi.12478 25807991

[27] Nance HA, Klimley P, Galván-Magaña F, Martínez-Ortíz J, Marko PB. Demographic Processes Underlying Subtle Patterns of Population Structure in the Scalloped Hammerhead Shark *Sphyrna lewini*. PLoS One. 2011;6: e21459. doi: 10.1371/journal.pone.0021459 21789171 PMC3137562

[28] Kyne PM, Carlson JK, Ebert DA, Fordham S V., Bizzarro JJ, Graham RT, et al. The Conservation Status of North American, Central American and Caribbean Chondrichthyans. IUCN Species Survival Commission Shark Specialist Group. Vancouver, Canada; 2012. p. 156.

[29] Pacoureau N, Rigby CL, Kyne PM, Sherley RB, Winker H, Carlson JK, et al. Half a century of global decline in oceanic sharks and rays. Nature. 2021;589: 567–571. doi: 10.1038/s41586-020-03173-9 33505035

[30] FAO. Fisheries Management 1. Conservation and management of sharks. FAO Technical Guidelines for Responsible Fisheries. No. 4. Suppl. 1. FAO 37, Rome. 2000.

[31] Klimley AP. The determinants of sexual segregation in the scalloped hammerhead shark, *Sphyrna lewini*. Environ Biol Fishes. 1987;18: 27–40. doi: 10.1007/bf00002325

[32] Coiraton C, Amezcua F, Ketchum JT. New insights into the migration patterns of the scalloped hammerhead shark *Sphyrna lewini* based on vertebral microchemistry. Mar Biol. 2020;167: 1–18. doi: 10.1007/s00227-020-3668-0

[33] Salinas-de-León P, Hoyos-Padilla EM, Pochet F. First observation on the mating behaviour of the endangered scalloped hammerhead shark *Sphyrna lewini* in the Tropical Eastern Pacific. Environ Biol Fishes. 2017;100: 1603–1608.

[34] Quintanilla S, Gómez A, Mariño-Ramirez C, Sorzano C, Bessudo S, Soler G, et al. Conservation genetics of the scalloped hammerhead shark in the Pacific coast of Colombia. J Hered. 2015;106: 448–458. doi: 10.1093/jhered/esv050 26245780

[35] Daly-Engel TS, Seraphin KD, Holland KN, Coffey JP, Nance HA, Toonen RJ, et al. Global phylogeography with mixed-marker analysis reveals male-mediated dispersal in the endangered scalloped hammerhead shark (*Sphyrna lewini*). PLoS One. 2012;7. doi: 10.1371/journal.pone.0029986 22253848 PMC3254628

[36] Klimley AP, Nelson DR. Schooling of the scalloped hammerhead *Sphyrna lewini* in the Gulf of California. Fish Bull. 1981;79: 256–260.

[37] Bejarano-Alvarez M, Galvan-Magana F, Ochoa-Baez RI. Reproductive biology of the scalloped hammerhead shark *Sphyrna lewini* (Chondrichthyes: Sphyrnidae) off south-west Mexico. aqua Int J Ichthyol. 2011;17: 11.

[38] Zanella I, López-Garro A. Abundancia, reproducción y tallas del tiburón martillo *Sphyrna lewini* (Carcharhiniformes: Sphyrnidae) en la pesca artesanal de Golfo Dulce, Pacífico de Costa Rica. Rev Biol Trop. 2014;63: 307–317.

[39] Robles YA, Montes LA, Vega ÁJ. Caracterización de la captura de tiburones por la pesca artesanal en los manglares de David, Golfo de Chiriquí, Pacífico de Panamá. Tecnociencia. 2015;17: 11–30.

[40] Estupiñán-Montaño C, Cedeño-Figueroa LG, Galván-Magaña F. Hábitos alimentarios del tiburón martillo *Sphyrna lewini* (Griffith & Smith, 1834) (Chondrichthyes) en el Pacífico ecuatoriano. Rev Biol Mar Oceanogr. 2009;44. doi: 10.4067/s0718-19572009000200011

[41] Hoyos-Padilla E, Ketchum JT, Klimley A, Galván-Magaña F. Ontogenetic migration of a female scalloped hammerhead shark *Sphyrna lewini* in the Gulf of California. Anim Biotelemetry. 2014;2: 17. doi: 10.1186/2050-3385-2-17

[42] Gallagher AJ, Klimley AP. The biology and conservation status of the large hammerhead shark complex: the great, scalloped, and smooth hammerheads. Rev Fish Biol Fish. 2018;28: 777–794. doi: 10.1007/s11160-018-9530-5

[43] Ketchum JT, Hearn A, Klimley AP, Espinoza E, Peñaherrera C, Largier JL. Seasonal changes in movements and habitat preferences of the scalloped hammerhead shark (*Sphyrna lewini*) while refuging near an oceanic island. Mar Biol. 2014;161: 755–767. doi: 10.1007/s00227-013-2375-5

[44] Cerutti-Pereyra F, Moity N, Dureuil M, Ramírez-González J, Reyes H, Budd K, et al. Artisanal longline fishing the Galapagos Islands–effects on vulnerable megafauna in a UNESCO World Heritage site. Ocean Coast Manag. 2019;183: 104995. doi: 10.1016/j.ocecoaman.2019.104995

[45] Bessudo S, Soler GA, Klimley PA, Ketchum J, Arauz R, Hearn A, et al. Vertical and horizontal movements of the scalloped hammerhead shark (*Sphyrna lewini*) around Malpelo and Cocos Islands (Tropical Eastern Pacific) using satellite telemetry. Bull Mar Coast Res. 2016;40. doi: 10.25268/bimc.invemar.2011.40.0.133

[46] Ketchum JT, Hoyos-Padilla M, Aldana-Moreno A, Ayres K, Galvan-Magaña F, Hearn A, et al. Shark movement patterns in the Mexican Pacific: a conservation and management perspective. 1st ed. Advances in Marine Biology. Elsevier Ltd.; 2020. doi: 10.1016/bs.amb.2020.03.002 32456839

[47] Torres-Rojas YE, Páez Osuna F, Camalich J, Galván Magaña F. Diet and trophic level of scalloped hammerhead shark (*Sphyrna lewini*) from the Gulf of California and Gulf of Tehuantepec, México. Iran J Fish Sci. 2015;14: 767–785.

[48] Rosende-Pereiro A, Flores-Ortega JR, González-Sansón G, Corgos A. Stomach content and stable isotopes reveal an ontogenetic dietary shift of young-of-the-year scalloped hammerhead sharks (*Sphyrna lewini*) inhabiting coastal nursery areas. Environ Biol Fishes. 2020;103: 49–65. doi: 10.1007/s10641-019-00932-0 Stomach

[49] Galván-Magaña F, Polo-Silva C, Berenice Hernández-Aguilar S, Sandoval-Londoño A, Ruth Ochoa-Díaz M, Aguilar-Castro N, et al. Shark predation on cephalopods in the Mexican and Ecuadorian Pacific Ocean. Deep Res Part II Top Stud Oceanogr. 2013;95: 52–62. doi: 10.1016/j.dsr2.2013.04.002

[50] Snell HM, Stone PA, Snell HL. A summary of geographical characteristics of the Galapagos Islands. J Biogeogr. 1996;23: 619–624. doi: 10.1111/j.1365-2699.1996.tb00022.x

[51] Banks SJ. Ambiente Físico. Reserva Marina de Galápagos, Línea Base de la Biodiversidad. In: Danulat E, Edgar GJ, editors. Reserva Marina de Galápagos, Línea Base de la Biodiversidad. Galápagos, Ecuador: Charles Darwin Foundation and Galápagos National Park Service; 2002. pp. 22–29.

[52] Edgar GJ, Banks S, Fariña JM, Calvopiña M, Martínez C. Regional biogeography of shallow reef fish and macro-invertebrate communities in the Galapagos archipelago. J Biogeogr. 2004;31: 1107–1124. doi: 10.1111/j.1365-2699.2004.01055.x

[53] Kessler WS. The circulation of the eastern tropical Pacific: A review. Prog Oceanogr. 2006;69: 181–217. doi: 10.1016/j.pocean.2006.03.009

[54] Palacios DM. Seasonal patterns of sea-surface temperature and ocean color around the Galapagos: Regional and local influences. Deep Res Part II Top Stud Oceanogr. 2004;51: 43–57. doi: 10.1016/j.dsr2.2003.08.001

[55] Wessel P, Smith WHF. A global, self-consistent, hierarchical, high-resolution shoreline database. J Geophys Res. 1996;101: 8741–8743. doi: 10.1029/96JB00104

[56] Olin JA, Hussey NE, Fritts M, Heupel MR, Simpfendorfer CA, Poulakis GR, et al. Maternal meddling in neonatal sharks: Implications for interpreting stable isotopes in young animals. Rapid Commun Mass Spectrom. 2011;25: 1008–1016. doi: 10.1002/rcm.4946 21452377

[57] Duncan KM, Holland KN. Habitat use, growth rates and dispersal patterns of juvenile scalloped hammerhead sharks *Sphyrna lewini* in a nursery habitat. Mar Ecol Prog Ser. 2006;312: 211–221. doi: 10.3354/meps312211

[58] Páez-Rosas D, Suarez-Moncada J, Elorriaga-Verplancken FR, Proaño A, Arnés-Urgellés C, Salinas-de-León P, et al. Trophic variation during the early stages of blacktip sharks (Carcharhinus limbatus) within coastal nurseries of the Galapagos Marine Reserve. J Sea Res. 2021;170. doi: 10.1016/j.seares.2021.102023

[59] Páez-Rosas D, Insuasti-Zarate P, Riofrío-Lazo M, Galván-Magaña F. Feeding behavior and trophic interaction of three shark species in the Galapagos Marine Reserve. Robertson DR, editor. PeerJ. 2018;6: e4818. doi: 10.7717/peerj.4818 29844971 PMC5971838

[60] Post D, Layman C, Arrington D, Takimoto G, Quattrochi J, Montaña C. Getting to the fat of the matter: models, methods and assumptions for dealing with lipids in stable isotope analyses. Oecologia. 2007;157: 179–189. 10.1007/s00442-006-0630-x 17225157

[61] Logan J, Jardine T, Miller T, Bunn S, Cunjak R, Lutcavage M. Lipid corrections in carbon and nitrogen stable isotope analyses: comparison of chemical extraction and modelling methods. J Anim Ecol. 2008;77: 838–846. doi: 10.1111/j.1365-2656.2008.01394.x 18489570

[62] Jackson A, Inger R, Parnell A, Bearhop S. Comparing isotopic niche widths among and within communities: SIBER—Stable Isotope Bayesian Ellipses. J Anim Ecol. 2011;80: 595–602. doi: 10.1111/j.1365-2656.2011.01806.x 21401589

[63] M T, M C. Food-web structure and fractionation of carbon isotopes in the Bering Sea. Mar Biol. 1979;53: 257–262.

[64] Michener R, Schell D. Stable isotope ratios as tracers in marine aquatic food webs. Black-Well. In: Lajth K, Michener R, editors. Stable isotopes in ecology and environmental science. Black-Well. Boston: Wiley and Blackwell; 1994. pp. 238–282.

[65] Altabet MA. Nitrogen isotopic evidence for micronutrient control of fractional NO3 equatorial Pacific. Limnol Oceanogr. 2001;46: 368–380.

[66] Sigman D, Granger J, DiFiore P, Lehmann M, Ho R, Cane G, et al. Coupled nitrogen and oxygen isotope measurements of nitrate along the eastern North Pacific margin. Global Biogeochem Cycles. 2005;19. doi: 10.1029/2005GB002458

[67] Loor-Andrade P, Galván-Magaña F, Elorriaga-Verplancken FR, Polo-Silva C, Delgado-Huertas A. Population and individual foraging patterns of two hammerhead sharks using carbon and nitrogen stable isotopes. Rapid Commun Mass Spectrom. 2015;29: 821–829. doi: 10.1002/rcm.7169 26377010

[68] Estupiñán-Montaño C, Galván-Magaña F, Sánchez-González A, Elorriaga-Verplancken FR, Delgado-Huertas A, Páez-Rosas D. Dietary ontogeny of the blue shark, *Prionace glauca*, based on the analysis of δ13C and δ15N in vertebrae. Mar Biol. 2019;166. doi: 10.1007/s00227-019-3550-0

[69] Torres-Rojas YE, Páez-Osuna F, Hernández-Herrera A, Galván-Magaña F, Aguiñiga-García S, Villalobos-Ortíz H, et al. Feeding grounds of juvenile scalloped hammerhead sharks (*Sphyrna lewini*) in the south-eastern Gulf of California. Hydrobiologia. 2014;726: 81–94. doi: 10.1007/s10750-013-1753-9

[70] Malpica-Cruz L, Herzka SZ, Sosa-Nishizaki O, Lazo JP. Tissue-specific isotope trophic discrimination factors and turnover rates in a marine elasmobranch: Empirical and modeling results. Can J Fish Aquat Sci. 2012;69: 551–564. doi: 10.1139/F2011-172

[71] Páez-Rosas D, Riofrío-Lazo M, Ortega J, de Dios Morales J, Carvajal R, Alava JJ. Southern elephant seal vagrants in Ecuador: a symptom of La Niña events? Mar Biodivers Rec. 2018;11: 13. doi: 10.1186/s41200-018-0149-y

[72] Vaudo J, Matich P, Heithaus R. Mother–offspring isotopes fractionation two species if placentatrophic sharks. J Fish Biol. 2010;77: 1724–1727. doi: 10.1111/j.1095-8649.2010.02813.x 21078031

[73] McMeans B, Olin J, Benz G. Stable isotope comparisons between embryos and mothers of a placentatrophic shark species. J Fish Biol. 2009;75: 2464–2474. doi: 10.1111/j.1095-8649.2009.02402.x 20738502

[74] Hamlett W. Sharks, skates and rays: the biology of elasmobrach fishes. Baltimore, Maryland. USA: The John Hopkins University Press; 1999.

[75] Llerena-Martillo Y. Identificación de tiburones juveniles y caracterización de sus hábitats en las zonas costeras de pesca de la isla San Cristóbal—Reserva Marina de Galápagos. Universidad de Guayaquil—BSc thesis. 2009.

[76] Chiriboga Y. Ecología espacial y conservación de tiburones neonatos y juveniles punta negra (*Carcharhinus limbatus*) en la Isla San Cristóbal–Reserva Marina de Galápagos. Universidad de San Francisco de Quito- BSc thesis. 2018.

[77] Llerena-Martillo Y, Peñaherrera-Palma C, Espinoza ER. Fish assemblages in three fringed mangrove bays of Santa Cruz Island, Galapagos Marine Reserve. Rev Biol Trop. 2018;66: 674–687.

[78] Pak H, Zanveld J. The Cromwell Current on the east side of the Galapagos. Geophys Res. 1973;78: 4845–7859.

[79] Bidigare R, Fluegge A, Freeman K, Hanson K, Hayes J, Hollander H, et al. Consistent fractionation of 13C in nature and in the laboratory: growth-rate effects in some haptophyte algae. Global Biogeochem Cycles. 1997;11: 279–292. doi: 10.1029/96gb03939 11540616

[80] Schell D, Barnett B, Vinette K. Carbon and nitrogen isotope ratios in zooplankton of the Bering, Chukchi and Beaufort Seas. Mar Ecol Prog Ser. 1998;162: 11–23.

[81] Fierro-Arcos D, Marín Jarrín J, Aburto-Oropeza O, Harvey E, Rastoin-Laplane E, Salinas-de-León P. Mangrove fish assemblages reflect the environmental diversity of the Galapagos Islands. Mar Ecol Prog Ser. 2021;664: 183–205. doi: 10.3354/meps13628

[82] Zanella I, López A, Arauz R. Caracterización de la pesca del tiburón martillo *Sphyrna lewini*, en la parte externa del Golfo de Nicoya, Costa Rica. Rev Ciencias Mar y Costeras. 2009;1: 175.

